# The effect of Moringa oleifera capsule in increasing breastmilk volume in early postpartum patients: A double-blind, randomized controlled trial

**DOI:** 10.1371/journal.pone.0248950

**Published:** 2021-04-06

**Authors:** Siraphat Fungtammasan, Vorapong Phupong

**Affiliations:** Placental Related Diseases Research Unit, Department of Obstetrics and Gynecology, Faculty of Medicine, Chulalongkorn University, Bangkok, Thailand; RCSI & UCD Malaysia Campus (formerly Penang Medical College), MALAYSIA

## Abstract

Moringa oleifera is an herbal galactagogue that has been used to increase the volume of breastmilk. Few studies have evaluated the effect of Moringa oleifera in breastfeeding. There are conflicting data whether it can increase the volume of breastmilk or not. Thus, the objective of this study is to evaluate the efficacy of Moringa oleifera leaves in increasing the volume of breastmilk in early postpartum mothers. A randomized, double-blind, placebo-controlled trial will be conducted. The outcomes of this study will provide the data of Moringa oleifera as an herbal medication to increase the volume of breastmilk. This information will be used to increase the rate of exclusive breastfeeding for the first 6 months as recommended by the World Health Organization.

Clinical trial registration

This clinical trial was registered at ClinicalTrials.gov (Clinical trials registration: NCT04487613).

## Introduction

Breastmilk is the best food for the baby. It is safe, clean and contains antibodies which protect them against common illnesses. It also contains good nutrients and energy for the baby, especially in the first month of life. Breastfeeding provides physiological and health benefits for both the mother and the baby. World Health Organization (WHO) and United Nations International Children’s Emergency Fund (UNICEF) recommend that the baby be breastfed within the first hour of life and exclusively for the first 6 months of life. WHO actively promotes breastfeeding as the best source of nourishment for infants and young children, and has set the rate of exclusive breastfeeding for the first 6 months up to at least 50% by year 2025 [1].

Adequate volume of breast milk is the factor for success in exclusive breastfeeding. Various methods have been used to increase the volume of breast milk. From the Cochrane database systemic review in 2020, demonstrated that natural milk boosters may improve milk volume and infants’ weight, but they are very uncertain about the supporting evidence. More high-quality studies are needed to increase the certainty about the effects of milk boosters [2]. Galactagogue herbs have been used by breastfeeding mothers who have breastmilk problem to increase the volume of breastmilk [3]. Moringa oleifera is an herbal galactagogue. Moringa oleifera is one of the herbs that has been used to increase the volume of breastmilk. Moringa oleifera are widely used in traditional medicine. Moringa oleifera leaves and immature seed pods are used as food products [4]. Moringa oleifera leaves increases breastmilk volume by increasing prolactin and providing essential nutrients [2, 5]. It takes about 24 hours after ingestion for the Moringa oleifera to work [6, 7]. Various safety studies were conducted in animals using aqueous leaf extracts and the results indicated that there was a high degree of safety. No adverse effects were reported in human studies [4]. There have been few studies that have evaluated Moringa oleifera in breastfeeding. One study found that the consumption of moringa cookies increased the quality of breastmilk, especially the amount of protein [8]. Another study found that Moringa oleifera leaves increased the production of breastmilk on postpartum days 4 to 5 among mothers who delivered preterm infants [7]. One study found that women who took Moringa oleifera capsules had more breastmilk per day from postpartum days 3 to 10 compared to those women who were on placebo. However, this was not statistically significant [6]. Thus, the objective of this study is to evaluate the efficacy of Moringa oleifera leaves in increasing the volume of breastmilk in early postpartum mothers.

## Materials and methods

This randomized, double-blinded, placebo-controlled trial will be performed at the Department of Obstetrics and Gynecology, King Chulalongkorn Memorial Hospital, Faculty of Medicine, Chulalongkorn University, Bangkok, Thailand. This is a baby friendly hospital. This study was approved by the Research Ethics Committee of the Faculty of Medicine, Chulalongkorn University (IRB No. 157/63). The study will be performed in accordance with the approved guidelines of the Research Ethics Committee. Written informed consent will be obtained from all participants. This clinical trial was registered at ClinicalTrials.gov (Clinical trials registration: NCT04487613). The authors confirm that all ongoing and related trials for this drug are registered. The complete date range for participant recruitment and follow-up will be 1 year and 6 months (November 1, 2020 –April 30, 2022).

Pregnant women aged 18 years or more and gestational age 37 weeks or more who intend to breastfeed will be invited to join this study. Recruitment is done and consent is obtained before delivery. Randomization will be done after delivery. Women with uncomplicated full term delivery who have accomplished similar antenatal breastfeeding promotion protocol will be included. Postpartum women with contraindication to breastfeeding such as HIV, on chemotherapeutic drugs, on radioactive substances, baby with galactosemia, postpartum women with unstable conditions (i.e., postpartum hemorrhage, sepsis), known allergy to Moringa oleifera, women whose baby need phototherapy, women with insufficient glandular tissue or breast surgery, women with a history of infertility, women with hypothyroidism, women with twins or higher order births, premature infants and infants with sucking problems or structural oral anomalies that can affect sucking (e.g. tongue tie, birth asphyxia, clefts, etc.) will be excluded.

After the study is approved, eligible consecutive postpartum women during the study period who gave informed consent will be enrolled into the study. All women will receive the same postpartum care along with breastfeeding support procedures. The research nurses confirm that all women correctly nursed their infants. Breastfeeding will be initiated immediately after delivery in all women. All women will be encouraged to breastfeed their baby as frequently as they desired or whenever the baby becomes hungry. The babies will be fed directly from the breasts. All women will be exclusively breastfeeding, and their baby will not be given any supplemental feeds. If the baby shows signs of inadequate milk intake, and if supplemental feeds are given, these are controlled for in the analysis. The data about supplemental feeds will be recorded from asking the women.

The drugs and placebo will be prepared prior to the study by a pharmacist who is not involved in the study. For the treatment capsule, it will contain 450 mg of Moringa oleifera leaves. As for the placebo capsule, there will be no content in the capsule. The participants will be asked if they think they got the placebo or the intervention to check on how effective the blinding was.

The participants will be randomized into two groups: treatment or placebo groups. A randomization scheme will be generated by a random number table using a block-of-four technique. The co-investigator, who has no contact with the participants, will generate the allocation sequence prior to the study. To ensure randomization, each envelope will be distributed in a sequential numerical order. Both the health care providers and the participants will be masked to the treatment assignment.

The nurses will enroll the participants. As soon as the participant meets the inclusion criteria, the nurses will proceed to select a sequentially numbered opaque envelope. The opaque envelopes will contain 6 capsules of Moringa oleifera leaves powder or placebo (identical in size, shape, and color). The opaque envelopes are sealed to ensure the allocation sequence is secure. The treatment group will receive Moringa oleifera leaves powder (450mg per capsule) (Ouay Un Osoth, Thailand) and the placebo group will receive no drug capsules. All women will take 1 capsule of the Moringa oleifera or placebo 2 times before meal for 3 days. Participants will take their first capsule at first 6 hours of birth. Treatment assignment will not be revealed until data collection is completed 6 months later. All women will be admitted into the postpartum ward and discharged on the fourth day postpartum. Hence, all women will receive all of their medications. The research nurses will capture the measures outcome and 8 personnel involve in this study. The dosage of 450 mg Moringa oleifera leaves powder is used in the study due to the recommendation from Thai traditional medicine for galactagogue. This dosage is higher than previous studies [6, 7].

**Fig 1:**
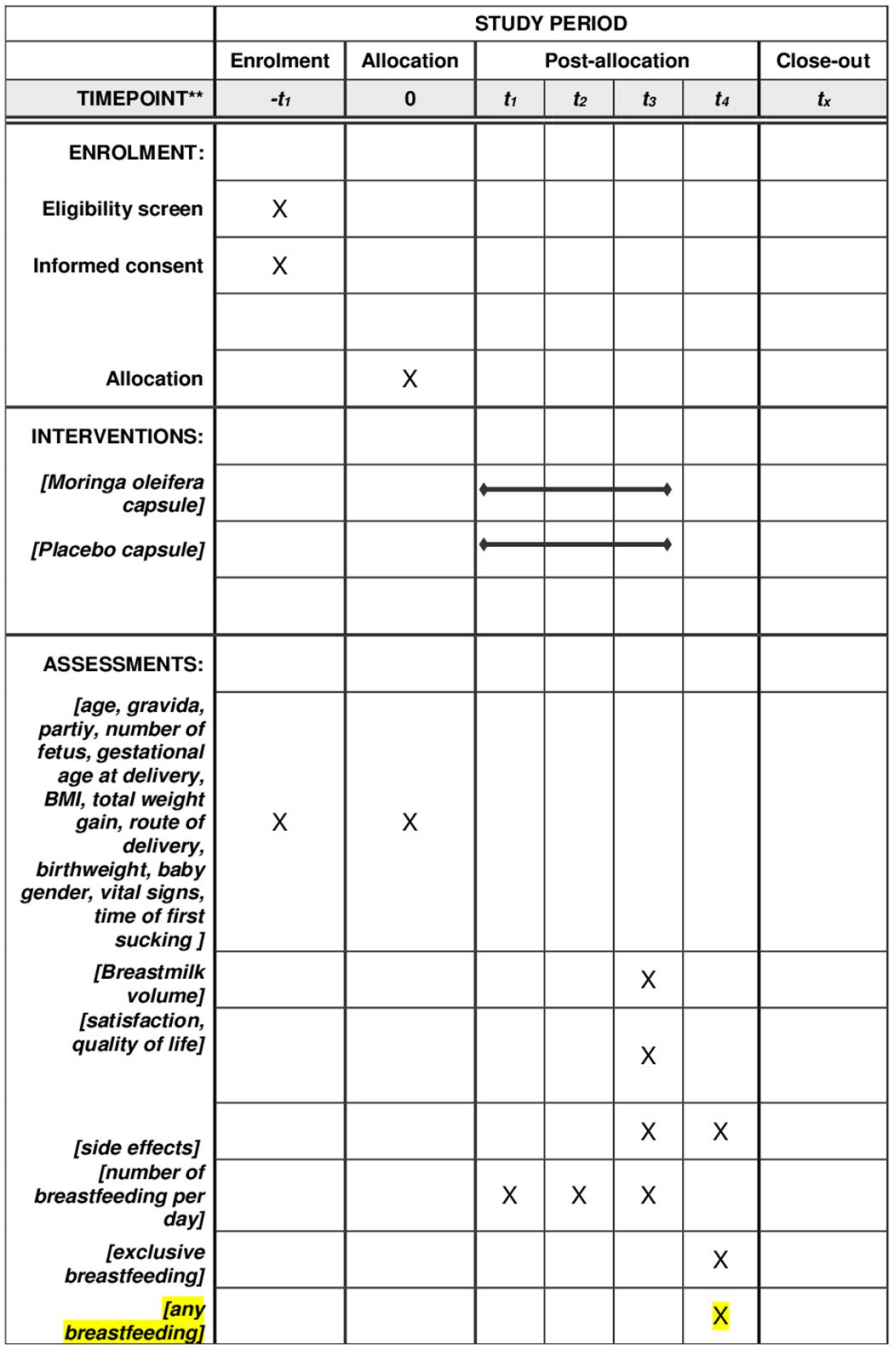
Recommended content for the schedule of enrolment, interventions, and assessments. *Recommended content can be displayed using various schematic formats. See SPIRIT 2013 Explanation and Elaboration for examples from protocols. **List specific timepoints in this row. *t*_*1*_: postpartum day 1, *t*_*2*_: postpartum day 2, *t*_*3*_: postpartum day 3, *t*_*4*_: six months postpartum.

The primary outcome is the volume of breastmilk on the third day postpartum. Secondary outcomes will assess the delta daily milk volume of consecutive 3 daily postpartum, time to noticeable breast fullness, the maternal satisfaction, quality of life, side effects, and the exclusive breastfeeding rates at 6 months (Fig 1). The third day is used as the measurement time point because it represents the timing of stage II lactogenesis [6]. The weighing method will be used on the third day postpartum (48–72 hour). The weighing procedure will start at 48 hours after delivery in all women. The nurse will weigh the infant fully clothed before and after each feeding using an electronic weight scale (Camry ER 7210, accurate to 5 g) for the period of 24 hours. The numbers of times weight measures taken will be 8–12 depending on frequency of breastfeeding of each participant. The baby would be weighed each time she/he wants to feed, even if it’s a baby who wants the breasts every 20 minutes.

The volume of the breastmilk will be evaluated. The sum of the weight difference in gram will be converted into the volume of the breastmilk in milliliter (1 g = 1mL). This method is comparable with the measurement of the volume of the breastmilk based on deuterium oxide dilution technique from a previous study [9–11].

Time to noticeable breast fullness is defined as the mean time from birth to noticeable breast fullness. The participants will be asked if they noticed their breasts were full and followed by ‘When did you feel breast fullness?’.

Satisfaction and quality of life will be asked at postpartum day 7 via a phone interview. Satisfaction answer choices will consist of the following: very satisfied, satisfied, neutral, unsatisfied, and very unsatisfied. Quality of life will be assessed by WHOQoL-BREF [12]. The participants will be asked with “Did the capsules help?”. Side effects are recorded at postpartum day 3 by interview and at postpartum day 7 via a phone interview. The exclusive breastfeeding rates and any breastfeeding at 6 months will be asked via a phone interview.

The sample size calculation is based upon the findings from a previous study [13]. Two-tailed test is used. The average volume of breastmilk on 3 days postpartum was 135.0 ± 61.5 mL. We expect that there will be a 30% increase in the volume of the breastmilk. With adjustments for a withdrawal rate of 20%, a minimum of 44 women in each group are required to detect statistical difference (α = 0.05, β = 0.2) between the two groups. Therefore, a total of 88 women are required for this study.

Data monitoring committee (DMC) is not needed in this study due to a short duration of the trial and known minimal risks. Interim analyses will not be performed in this trial due short duration of recruitment and no potentially serious outcomes. No adverse effects were reported in human studies. However, the risk and harm are monitored. Both type of risk and severity are monitored. Side effects (such as constipation, nausea/vomiting, diarrhea, heartburn, hypotension, hypoglycemia) and serious adverse effects/serious adverse reactions both in mother and especially vulnerable newborn (such as neonatal hypoglycemia, hypotension) are monitored. Harm will be monitored and report to Research Ethics Committee of the Faculty of Medicine, Chulalongkorn University. Auditing will be performed by research nurses who are not involved in the study every 3 months. Investigators plan to provide care for participants’ healthcare needs that arise as a direct consequence of trial participation and pay for compensation to those who suffer harm from trial participation.

Principal investigators will have access to the final trial dataset. Investigators plan to communicate trial results to participants and send for publication in peer reviewed journal.

### Statistical analysis

**Fig 2:**
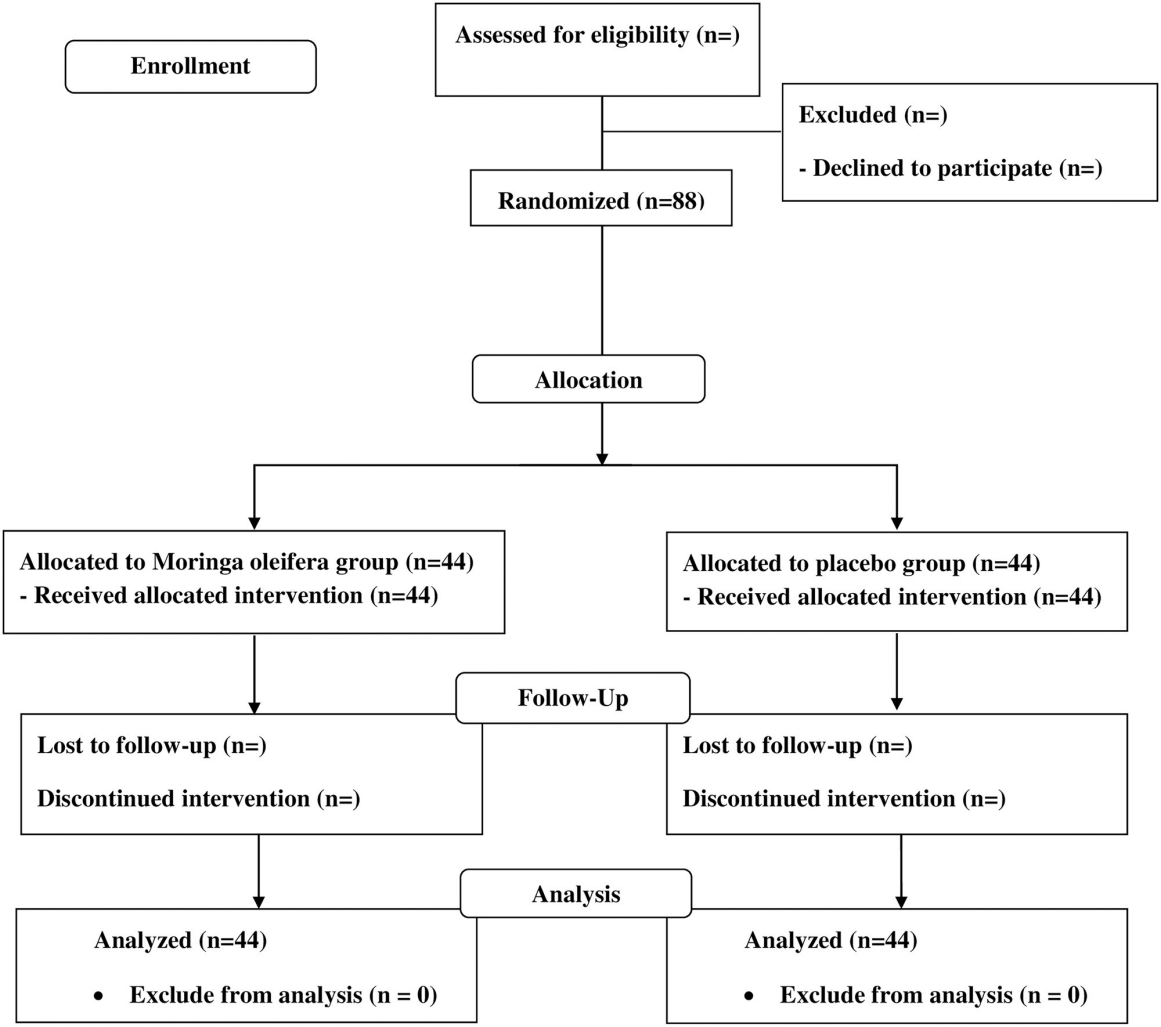
CONSORT flowchart.

IBM SPSS version 22 (SPSS: An IBM Company, New York, USA) will be used for statistical analysis. Two-tailed test is used in this study. Kolmogorov-Smirnov tests will be used to assess data distribution prior to statistical analysis. Chi-square test and Fisher’s exact test will be used for categorical variables such as percentage of satisfaction and side effects. Independent t-test will be used for parametric continuous variables such as the volume of the breastmilk. Mann-Whitney U test will be used for nonparametric variables when appropriate. A p-value <0.05 will be considered statistically significant. Analysis of the trial will be conducted by using intent-to-treat (ITT) analysis (Fig 2).

## Supporting information

S1 File (DOC)

S2 File (DOC)

S3 File (SAV)

S1 Checklist SPIRIT 2013 checklist: Recommended items to address in a clinical trial protocol and related documents*. (DOC)

S1 Table Baseline characteristics. (DOC)

S2 Table Breast milk volume, satisfaction, quality of life and side effects. (DOCX)
