# Correction: The effect of Moringa oleifera capsule in increasing breastmilk volume in early postpartum patients: A double-blind, randomized controlled trial

**DOI:** 10.1371/journal.pone.0350254

**Published:** 2026-05-27

**Authors:** The PLOS One Editors

After publication of this Registered Report Protocol [1], concerns were raised about the study dates reported in the Materials and Methods section. Specifically, the study dates (participant recruitment and follow‑up: November 1, 2020 – April 30, 2022) preceded the Registered Report Protocol’s acceptance date of March 7, 2021. This conflicts with Registered Report requirements, which indicate that study acceptance must occur before the research begins. The corresponding author VP indicated that the reported start date is incorrect, and represents a planned start date that was included in the initial submission, with the intention to begin only after acceptance.

The correct study start date is April 1, 2021. Corresponding author VP provided documentation issued by an institutional representative at Chulalongkorn University (Bangkok, Thailand) in support of this information.

## References

[1] Fungtammasan S, Phupong V. The effect of Moringa oleifera capsule in increasing breastmilk volume in early postpartum patients: A double-blind, randomized controlled trial. PLoS One. 2021;16(4):e0248950. doi: 10.1371/journal.pone.0248950 33822798 PMC8023461

[2] PLOS One. Submission Guidelines [Internet]. [cited 2026 May]. Available from: https://journals.plos.org/plosone/s/submission-guidelines#loc-registered-reports

