# Supplementary material for: The effect of Moringa oleifera capsule in increasing breastmilk volume in early postpartum patients: A double-blind, randomized controlled trial
Source: PLoS One. 2021 Apr 6;16(4):e0248950. doi: 10.1371/journal.pone.0248950 (PMC8023461; doi:10.1371/journal.pone.0248950)
Supplement: S1 File — (DOC) [file pone.0248950.s001.doc]

โครงร่างการวิจัย (Research Proposal)

1. ชื่อโครงการ (Proposal Title)

การทดลองแบบสุ่ม แบบปิดบังทั้งสองทางและมีกลุ่มควบคุม เพื่อศึกษาผลของแคปซูลใบมะรุมในการเพิ่มปริมาณน้ำนมในหญิงหลังคลอด

Effect of Moringa oleiferaleafcapsulein Increasing Breast Milk Volume in Early Postpartum Patients, A Double blind, Randomized Controlled Trial

1. ชื่อคณะผู้วิจัย (Investigators)

ผู้วิจัยหลัก พญ.สิรภัทร ฟุ้งธรรมสาร

สังกัด ภาควิชาสูติศาสตร์ - นรีเวชวิทยา คณะแพทยศาสตร์ จุฬาลงกรณ์มหาวิทยาลัย

ความรับผิดชอบต่อโครงการวิจัย ผู้ทำวิจัย

ผู้วิจัยร่วม ศ.นพ.วรพงศ์ ภู่พงศ์

สังกัด ภาควิชาสูติศาสตร์ - นรีเวชวิทยา คณะแพทยศาสตร์ จุฬาลงกรณ์มหาวิทยาลัย

ความรับผิดชอบต่อโครงการวิจัย ที่ปรึกษาการวิจัย

1. ความสำคัญและที่มาของปัญหา (Rationale)

- น้ำนมจากมารดาถือเป็นอาหารที่เหมาะสมกับทารกในวัยตั้งแต่แรกเกิดจนถึงอายุ 6 เดือนที่ดีที่สุด ที่ยังไม่สามารถหาอาหารชนิดอื่นมาทดแทนได้ รวมถึงการให้นมจากเต้านมเป็นการสร้างเสริมความผูกพันระหว่างทารกและมารดา และส่งผลดีกับสุขภาพของมารดาในหลากหลายแง่มุมแต่การให้นมบุตรนั้นอาจเป็นเรื่องที่ทำได้ยากสำหรับมารดาหลังคลอด และมีอุปสรรคที่อาจเกิดขึ้นทำให้ลดการให้นมบุตรจากมารดา หนึ่งในอุปสรรคเหล่านั้นคือความรู้สึกว่ามารดาไม่สามารถให้น้ำนมที่เพียงพอกับบุตรได้ ทำให้ขาดความมั่นใจในการให้นมบุตร^(1),(2)^และรู้สึกว่าตนเองนั้นมีปริมาณน้ำนมที่ไม่เพียงพอสุดท้ายส่งผลให้เลิกการให้นมบุตรและเปลี่ยนไปใช้นมผสมแทนซึ่งสะดวกกว่าและสามารถกำหนดปริมาณที่ต้องการได้ง่ายกว่า การมียาที่จะสามารถเพิ่มปริมาณน้ำนมได้จะเป็นตัวช่วยส่งเสริมให้เพิ่มอัตราการให้นมบุตรได้มากขึ้น และเสริมสร้างความมั่นใจให้กับมารดาหลังคลอดได้ แต่อีกปัจจัยที่อาจทำให้เกิดความวิตกกังวลของมารดาที่ให้นมบุตรคือ ยาเหล่านั้นที่ใช้ในการเพิ่มปริมาณน้ำนมนั้น มีผลข้างเคียงที่ไม่พึงประสงค์ ที่บุตรของตนอาจจะได้รับหรือไม่ การใช้ยาสมุนไพรเพื่อเพิ่มปริมาณน้ำนมอาจเป็นตัวเลือกหนึ่งของมารดาที่ให้นมบุตรทดแทนยาแผนปัจจุบัน ด้วยความรู้สึกว่าเป็นสิ่งที่ได้จากธรรมชาติน่าจะมีความปลอดภัยมากกว่ายาที่สังเคราะห์ขึ้นมา^(3),(4)^รวมไปถึงมีผลข้างเคียงที่ไม่พึงประสงค์น้อยกว่าและสามารถหาซื้อได้ง่าย ยาสมุนไพรที่มีสรรพคุณในการเพิ่มน้ำนมที่สามารถหาซื้อได้ทั่วไปในประเทศไทยมีหลากหลายชนิด^(5)^ เช่น ขิง^(6)^ ดีปลี กระชาย และใบมะรุม^(7),(8),(9)^ซึ่งเป็นพืชที่สามารถพบได้มากในประเทศไทย แต่อย่างไรก็ตาม ในปัจจุบันมีการศึกษาที่เกี่ยวกับยาสมุนไพรที่ช่วยเพิ่มปริมาณน้ำนมไม่มากนัก และยังไม่มีการศึกษาที่พิสูจน์ได้ว่ายาเหล่านั้นสามารถเพิ่มปริมาณน้ำนมได้จริงจึงเป็นที่มาของปัญหาที่ทำให้เกิดงานวิจัยชิ้นนี้ขึ้น

1. ทบทวนวรรณกรรมงานวิจัยที่เกี่ยวข้อง (Literature Review)

- จากการศึกษาของ Peter Francis N^(7)^ที่ประเทศฟิลิปปินส์ ในปี2014รวบรวมการศึกษาเกี่ยวกับคุณสมบัติในการเพิ่มปริมาณน้ำนมของใบมะรุมพบว่ากลไกหลักที่ทำให้เพิ่มปริมาณน้ำนมได้คือการทำให้ระดับ prolactin level สูงขึ้น โดยพบว่าคนที่ได้รับยาแคปซูลใบมะรุมมีระดับ prolactin ที่สูงขึ้นกว่าคนที่ไม่ได้รับอย่างมีนัยสำคัญทางสถิติ
- การศึกษาโดย Janet Frawley et al.^(3)^ที่ประเทศออสเตรเลียและ การศึกษาของ Nursyuhadah Othman et al^(4)^. ที่ประเทศมาเลเซียพบว่ามีปริมาณหญิงตั้งครรภ์และให้นมบุตรจำนวนมากที่ใช้ยาสมุนไพรเพื่อหวังผลในการลดอาการระหว่างตั้งครรภ์ และการเพิ่มน้ำนมบุตรขณะให้นมบุตร ด้วยความเชื่อที่ว่ายาสมุนไพรนั้นได้มาจากธรรมชาติ น่าจะมีผลข้างเคียงกับบุตรน้อยกว่าและปลอดภัยกว่ายาแผนปัจจุบัน และยาสมุนไพรสามารถหาซื้อได้ง่าย จึงทำให้มีการใช้ยาสมุนไพรในหญิงทั้ง 2 กลุ่มนี้เพิ่มขึ้นในปัจจุบัน
- การศึกษาโดยCorazon P. Estrella ^(8)^ปี 2000เป็นการศึกษาแบบ Randomized Controlled Trial เพื่อเปรียบเทียบผลของใบมะรุมในการเพิ่มการหลั่งน้ำนมในหญิงตั้งครรภ์ที่คลอดบุตรก่อนครบกำหนด (น้อยกว่า37 สัปดาห์)และทารกได้รับการรักษาในหอผู้ป่วยวิกฤตของทารกแรกเกิด (NICU)ขนาดยาที่ใช้คือ250 mgรับประทานทุก 12 ชั่วโมง เริ่มรับประทานตั้งแต่วันที่ 3 หลังคลอด วัดผลโดยการวัดปริมาณน้ำนมจากการปั๊มน้ำนมใส่ขวด ทุก 4 ชั่วโมง ในวันที่ 3-5 หลังคลอด พบว่าปริมาณน้ำนมในมารดาที่ได้รับยา มีปริมาณมากกว่ากลุ่มที่ไม่ได้รับยาทั้งวันที่ 3,4 และ 5 โดยในวันที่ 5 นั้น ความแตกต่างของปริมาณน้ำนมมากที่สุด และไม่พบว่ามีผลข้างเคียงจากการได้รับยาชนิดนี้
- การศึกษาโดยCriselda L. Espinosa Kuo ^(9)^ ในปี2005เป็นการศึกษาแบบ Single blind randomized controlled trialเป็นการศึกษาผลของใบมะรุมในการเพิ่มปริมาณน้ำนมในหญิงที่คลอดทารกครบกำหนดทางช่องคลอดเทียบกับยาหลอกโดยใช้แคปซูลใบมะรุมขนาด 350 มิลลิกรัม 2 เม็ด วันละ 1 ครั้ง โดยเริ่มรับประทานตั้งแต่วันที่ 3 จนถึงวันที่ 10 หลังคลอด จากนั้นทำการวัดปริมาณน้ำนมโดยการปั๊ม พบว่าในกลุ่มที่ได้รับแคปซูลใบมะรุมนั้น มีปริมาณน้ำนมที่มากกว่ากลุ่มที่ได้รับยาหลอก แต่ไม่พบว่ามีนัยสำคัญทางสถิติ รวมถึงไม่พบว่ามีผลข้างเคียงใดเกิดขึ้นจากการใช้ยาชนิดนี้
- การศึกษาของPanwaraParitakul et al.^(6)^เป็น Randomized Controlled trial ศึกษาผลของแคปซูล Iขิงในการเพิ่มปริมาณน้ำนมเทียบกับยาหลอก โดยวัดในวันที่3 หลังคลอดซึ่งเหตุผลในการเลือกวัดในวันที่ 3 คือเป็นเวลาที่เริ่มต้นของ stage II lactogenesis ซึ่งก็คือระยะที่เริ่มมีการหลั่งน้ำนมในปริมาณมากหลังทารกและรกคลอด ใช้วิธีวัดโดยการชั่งน้ำหนักทารกก่อนและหลังรับประทานนม และนำมาลบกันเพื่อหาผลต่าง โดยถือว่าผลต่างของน้ำหนักคือปริมาณน้ำนมที่สร้างได้ในแต่ละมื้อและนำมารวมกันตลอด24 ชั่วโมงพบว่าขิงสามารถเพิ่มปริมาณน้ำนมได้จริง และวัดระดับ serum prolactin level เทียบกันในแต่ละกลุ่มพบว่าไม่มีความแตกต่าง โดยกลไกที่ทำให้มีผลเช่นนี้คาดว่าเกิดจากการมีฤทธิ์ vasodilation และ เพิ่ม blood supply สู่ mammary glands
- การศึกษาของ Sidney J. Stohs et al^(10)^ในปี 2015 เรื่องtoxicity โดยการรวบรวมข้อมูลจากหลายการศึกษาเกี่ยวกับผลข้างเคียงจากการใช้ใบมะรุมทั้งในสัตว์ทดลองและมนุษย์พบว่าการใช้มะรุมในรูปแบบต่าง ๆ ทั้งในรูปแบบผง หรือสารสกัดด้วยน้ำ เมทานอลหรือเอทานอล ไม่ทำให้เกิดอาการเป็นพิษ ถึงแม้ว่าจะใช้ในขนาดที่สูงมากเกินกว่าขนาดที่ใช้บริโภคปกติ โดยพบว่าจะทำให้เกิดพิษได้นั้น ต้องรับประทานในปริมาณที่มากกว่าปริมาณที่ใช้บริโภคปกติมาก เช่นมีผล genotoxic ที่ขนาดยา 3000 มิลลิกรัมต่อกิโลกรัมของหนู อีกทั้งยังมีการศึกษาเกี่ยวกับสรรพคุณของใบมะรุมในมนุษย์ พบว่าการใช้ผงใบมะรุมขนาด 8จนถึง 50กรัมต่อวันสามารถช่วยลดระดับน้ำตาลในเลือดได้ รวมทั้งยังมีสรรพคุณในการลดระดับไขมันในเลือดและเป็น antioxidant โดยไม่ก่อให้เกิดผลข้างเคียงใดๆในมนุษย์
- จากงานวิจัยในปี 1988 โดย Nancy F Butte et al.^(11)^ ศึกษาเรื่องความแม่นยำในวิธีการวัดปริมาณน้ำนมที่ทารกได้รับโดยวิธีการชั่งน้ำหนักทารก เทียบกับวิธีหลังรับประทานนมเปรียบเทียบกับวิธีการใช้ Deuterium peroxide ซึ่งเป็นวิธีมาตรฐานในการวัดปริมาณน้ำนม โดยการให้มารดารับประทานน้ำที่มีองค์ประกอบทางเคมีเป็น ^2^H_2_O (Deuterium oxide) และให้ทารกรับประทาน^18^O 60 mg/kg เพื่อวัดปริมาณ Total body water ของทารก จากนั้นจึงนำปัสสาวะของทารกมาตรวจวิเคราะห์ทางเคมี ตรวจอัตราส่วนของ ^2^H:^1^H โดยวิธี gas-isotope-ratio mass spectrometry และอัตราส่วนของ ^18^O:^16^O จากหลักการที่ว่า ^2^H และ ^18^O เป็น isotope ของ H (Hydrogen) และ O (Oxygen) ตามลำดับนั้น มีปริมาณน้อยมากในธรรมชาติ ^2^H ที่ตรวจพบนั้นจะต้องมาจากมารดาเท่านั้น นำมาคำนวณเทียบกับปริมาณ Total body water ของทารก พบว่าทั้ง2 วิธีวัดปริมาณน้ำนมได้ใกล้เคียงกันอย่างมาก โดยมี mean difference เท่ากับ 12 g/d ซึ่งเทียบเท่ากับ difference ทางสถิติเท่ากับ 0 เพราะฉะนั้นจึงสามารถถือได้ว่า การชั่งน้ำหนักทารก ด้วยตราชั่งที่มาตรฐานจะสามารถแสดงถึงปริมาณน้ำนมที่ทารกได้รับได้อย่างแม่นยำ

1. วัตถุประสงค์ของการวิจัย (Objectives)

วัตถุปะสงค์(หลัก) :

- เพื่อศึกษาเปรียบเทียบปริมาณน้ำนมที่หญิงหลังคลอดสร้างได้ระหว่างกลุ่มที่ได้รับแคปซูลใบมะรุมและกลุ่มที่ได้รับยาหลอก

วัตถุประสงค์(รอง)

- เพื่อศึกษาความพึงพอใจคุณภาพชีวิต และผลข้างเคียงหลังจากได้รับยาในแง่ของการให้นมบุตรเปรียบเทียบระหว่างกลุ่มที่ได้รับแคปซูลใบมะรุมและยาหลอก

1. คำถามของการวิจัย (Hypothesis)

คำถาม(หลัก):

- ยาแคปซูลใบมะรุมสามารถเพิ่มปริมาณน้ำนมในหญิงหลังคลอดได้มากกว่าการรับประทานยาหลอก

คำถาม(รอง)

- ยาแคปซูลใบมะรุมสามารถเพิ่มความพึงพอใจ คุณภาพชีวิตในแง่ของการให้นมบุตรได้หรือไม่ และมีผลข้างเคียงอย่างไรบ้าง

1. คำสำคัญ (Keywords)

- Lactation
- Galactogogue
- Moringa oleifera
- Drumstick tree
- Breast milk volume
- Herbal medicine

1. รูปแบบการวิจัย (Research design)

- การทดลองแบบสุ่มโดยมีกลุ่มเปรียบเทียบ
- Randomized Controlled Trial
- Double blind

1. ระเบียบวิธีการวิจัย (Research Methodology)

- ประชากร (Population) : หญิงหลังคลอดบุตร ที่ไม่มีข้อห้ามสำหรับการให้นมบุตร และมีความตั้งใจจะให้นมบุตรทั้งในกลุ่มที่คลอดปกติและผ่าตัดคลอด
- ประชากรเป้าหมาย (Target Population) : หญิงหลังคลอดที่ไม่มีข้อห้ามสำหรับการให้นมบุตร อายุตั้งแต่ 18 ปีขึ้นไป ที่คลอดบุตรที่โรงพยาบาลจุฬาลงกรณ์ทั้งในกลุ่มที่คลอดปกติและผ่าตัดคลอด
- ประชากรกลุ่มควบคุม (Control Population) : หญิงหลังคลอดที่ไม่มีข้อห้ามสำหรับการให้นมบุตร อายุตั้งแต่ 18 ปีขึ้นไป ที่คลอดบุตรที่โรงพยาบาลจุฬาลงกรณ์ ที่ได้รับยาหลอก
- เกณฑ์การคัดเลือกอาสาสมัครเข้าร่วมโครงการวิจัย (Inclusion Criteria) :
  - หญิงหลังคลอดที่มีอายุตั้งแต่ 18 ปีขึ้นไป ที่มีความตั้งใจจะให้นมบุตรทั้งในกลุ่มที่คลอดปกติและกลุ่มที่ผ่าตัดคลอด
- เกณฑ์คัดเลือกอาสาสมัครออกจากโครงการวิจัย (Exclusion Criteria) :
  - หญิงหลังคลอดที่มีข้อห้ามในการให้นมบุตร ได้แก่
    - มีการใช้ยาเสพติด ยาผิดกฎหมายเช่นamphetamine, cocaine, heroin, marijuana, phencyclidine, alcohol, smoking
    - มีทารกเป็นโรค Galactosemia
    - มีการติดเชื้อ HIV,Human T cell leukemia virus, Infectious mononucleosis , Ebola virus,Marburg virus, Lassa virus, dengue virus, adenoviruses
    - เป็นโรควัณโรคและยังไม่ได้รับการรักษา
    - รับประทานยาเหล่านี้
      - Cyclophosphamide Cyclosporine Doxorubicin Methotrexate
      - Copper 64 (64Cu), Gallium 67 (67Ga), Indium 111 (111In), Iodine 123 (123I),Iodine 125 (125I), Iodine 131 (131I), Radioactive sodium, Technetium 99m (99mTc), Macroaggregates, sodium pertechnetate (99mTcO4)
  - หญิงหลังคลอดอยู่ในภาวะที่ไม่สามารถให้นมบุตรได้ ได้แก่ อยู่ในภาวะวิกฤต มีสัญญานชีพที่ไม่คงที่ไม่สามารถให้นมบุตรได้ มีภาวะตกเลือดหลังคลอด (Postpartum hemorrhage)
  - หญิงหลังคลอดที่มีประวัติแพ้มะรุม
  - หญิงหลังคลอดที่ทารกต้องได้รับการส่องไฟรักษาภาวะตัวเหลือง
  - หญิงหลังคลอดที่มีเนื้อเยื่อเต้านมน้อยไม่เพียงพอหรือเคยผ่าตัดเต้านม
  - หญิงหลังคลอดที่มีประวัติมีบุตรยาก
  - หญิงหลังคลอดที่มีภาวะไทรอยด์ฮอร์โมนต่ำ
  - หญิงหลังคลอดที่คลอดครรภ์แฝด, คลอดก่อนกำหนด และทารกมีปัญหาการดูดนมหรือมีความผิดปกติของปาก เช่น พังผืดที่ลิ้น การขาดอากาศขณะคลอด ปากแหว่งเพดานโหว่
- กระบวนการขอความยินยอม (Informed consent process)
  - แพทย์พยาบาลและเจ้าหน้าที่ประจำหอผู้ป่วยที่ไม่เกี่ยวข้องเป็นแพทย์ผู้ดูแลรักษาผู้ป่วย เพื่อไม่ให้เกิดภาวะบีบบังคับทำให้ผู้ป่วยตัดสินใจไม่เป็นอิสระ อธิบายข้อมูลเกี่ยวกับงานวิจัย อธิบายจุดประสงค์และผลของการวิจัย และเปิดโอกาสให้ผู้ที่สนใจได้สอบถามข้อสงสัยต่าง ๆวิธีการปฏิบัติตนหากเข้าร่วมการวิจัย ประโยชน์และความเสี่ยงที่อาจเกิดขึ้นให้กับผู้ที่สนใจเข้าร่วมงานวิจัย อาสาสมัครผู้มีความสนใจจะได้รับเอกสารข้อมูลเกี่ยวกับงานวิจัยเพื่อพิจารณาการเข้าร่วม และเปิดโอกาสให้ผู้ที่สนใจได้สอบถามข้อสงสัยต่าง ๆ ตอบข้อสงสัยจนผู้ป่วยที่ได้รับเชิญให้เข้าร่วมการวิจัยเข้าใจและให้เวลาตัดสินใจโดยอิสระ ณ หอผู้ป่วยที่อาสาสมัครอยู่ก่อนลงนามให้ความยินยอมเข้าร่วมในการวิจัย
- วิธีการวิจัย/วิธีดำเนินการวิจัย
  - เขียนโครงร่างงานวิจัย นำเสนอต่อคณะกรรมการจริยธรรมงานวิจัย คณะแพทยศาสตร์ จุฬาลงกรณ์มหาวิทยาลัย
  - หญิงตั้งครรภ์อายุ 18 ปีขึ้นไปและอายุครรภ์ตั้งแต่ 37 สัปดาห์ที่มีความต้องการจะให้นมบุตรจะถูกเชิญชวนให้เข้าร่วมในการวิจัย และลงนามให้ความยินยอมเข้าร่วมในการวิจัยซึ่งจะทำก่อนการคลอด ส่วนการสุ่มให้เข้ากลุ่มการศึกษาจะทำหลังคลอด
  - คัดเลือกอาสาสมัครเข้าร่วมงานวิจัย (Inclusion criteria) จากหญิงหลังคลอดที่ได้ทำการคลอดบุตรที่โรงพยาบาลจุฬาลงกรณ์ทั้งหญิงที่คลอดปกติและผ่าตัดคลอด และอายุตั้งแต่ 18 ปีขึ้นไป ที่มีความต้องการจะให้นมบุตร หญิงที่มีข้อห้ามในการให้นมบุตร หรืออยู่ในภาวะที่ไม่สามารถให้นมบุตรได้จะถูกคัดออกจากงานวิจัย (Exclusion criteria)
  - ผู้ที่สนใจจะเข้าร่วมโครงการจะได้รับคำอธิบายจากผู้ทำวิจัยเกี่ยวกับข้อมูลเกี่ยวกับโครงการ จุดประสงค์และผลของการวิจัย และเปิดโอกาสให้ผู้ที่สนใจได้สอบถามข้อสงสัยต่าง ๆ
  - ผู้ที่เข้าร่วมโครงการลงนามยินยอมในการเข้าร่วมโครงการวิจัย หลังจากที่ได้ตัดสินใจอย่างอิสระแล้ว
  - ทำการสุ่มด้วยวิธี Allocation by block of 4เพื่อควบคุม และกระจายปัจจัยกวนอื่น ๆ ที่อาจมีผลต่อปริมาณน้ำนมให้เท่ากันทั้งในกลุ่มทดลองและกลุ่มควบคุม
  - บันทึกข้อมูลของอาสาสมัครที่อาจเป็นปัจจัยกวนที่อาจมีผลต่อปริมาณน้ำนม ได้แก่จำนวนการคลอดที่ผ่านมา ปริมาณน้ำที่ได้รับเข้าในร่างกายและขับถ่ายออกในแต่ละวัน (Intake/Output) อุณหภูมิร่างกาย ปัจจัยเหล่านี้จะถูกนำมาใช้ในขั้นตอนการวิเคราะห์ผลของงานวิจัย
  - แบ่งกลุ่มอาสาสมัครเป็น2 กลุ่มโดยการสุ่ม ได้แก่กลุ่มที่ได้ยาแคปซูลมะรุม(กลุ่มทดลอง)และกลุ่มที่ได้รับยาหลอก(กลุ่มควบคุม) โดยที่กลุ่มทดลองจะได้รับยาแคปซูลใบมะรุมขนาดเม็ดละ 450 มิลลิกรัมครั้งละ 1 เม็ด ก่อนอาหารมื้อเช้าและมื้อเย็น (รวมเป็น 900 มิลลิกรัมต่อวัน) **อาสาสมัครจะได้รับประทานยาเม็ดแรกที่เวลา 6 ชั่วโมงหลังคลอด** อ้างอิงจากงานวิจัยของ Krishanu S^(12)^ที่ใช้ยาในขนาด 900 มิลลิกรัมต่อวัน เนื่องจากการใช้มะรุมนั้นยังไม่มีขนาดมาตรฐานจึงอ้างอิงขนาดยาจากการใช้ในข้อบ่งชี้อื่น และเป็นขนาดที่พบใช้ซื้อขายในท้องตลาดมากที่สุด สามารถหาซื้อมาบริโภคได้ทั่วไปโดยเม็ดยาแคปซูลใบมะรุมจะถูกครอบโดยแคปซูลสีทึบอีกหนึ่งชั้นโดยเภสัชกรผู้ช่วยในการวิจัย และกลุ่มควบคุมจะได้ยาหลอกซึ่งเป็นแคปซูลที่เปล่าลักษณะเดียวกันกับที่ใช้ครอบเม็ดยาจริงเพื่อให้ลักษณะภายนอกเหมือนกันโดยที่จะมีซองกระดาษทึบที่บรรจุยา เรียงเลขลำดับซองแบบดังที่กล่าวโดยเภสัชกรผู้ที่ไม่เกี่ยวข้องกับงานวิจัยนี้ แต่ละซองบรรจุแคปซูลใบมะรุมสำหรับรับประทานทั้งหมด 3 วัน(6 เม็ด)โดยเริ่มรับประทานตั้งแต่หลังคลอด จนถึงวันที่ 3 (ชั่วโมงที่ 72 หลังคลอด)
  - แคปซูลใบมะรุมที่ใช้ในงานวิจัยครั้งนี้เป็นผลิตโดยบริษัทเอกสมุนไพร ที่อยู่98/31 ซอยแฟคตอรี่แลนด์ 2 ซอย 3 ถนนพุทธมณฑลสาย 5 หมู่ 11 ตำบลไร่ขิง อำเภอสามพราน จังหวัดนครปฐมชื่อทางการค้าคือ มะรุมแคปซูล (ผลิตภัณฑ์เสริมอาหารใบมะรุม ตราเฮอร์เบิลวัน)จัดจำหน่ายโดยบริษัททอ้วยอันโอสถ ได้รับการับรองมาตรฐาน GMPPIC/S(Good Manufacturing Practice Pharmaceutical Inspection Cooperation Scheme) โดยสำนักงานคณะกรรมการอาหารและยาแห่งประเทศไทย ได้รับการขึ้นทะเบียนเป็นผลิตภัณฑ์เสริมอาหาร มีหมายเลขอย.คือ73-1-49541-1-0015 และยาหลอกเป็นแคปซูลเปล่าสีทึบแบบเดียวกับที่ใช้ครอบแคปซูลใบมะรุมจริง แต่ไม่มียาอยู่ด้านในแคปซูล โดยแคปซูลเปล่าที่ใช้เป็นยาหลอกนั้นจะถูกจัดเตรียมโดยเภสัชกรผู้ช่วยในการวิจัย
  - เก็บข้อมูลรวบรวมข้อมูลในวันที่3 (ชั่วโมงที่ 48-72หลังคลอด) โดยทำการชั่งน้ำหนักทารกทุกครั้งบนตาชั่งที่แสดงผลเป็นตัวเลขดิจิทัล มีจุดทศนิยม 2 ตำแหน่งก่อนและหลังการให้นมในแต่ละครั้ง โดยกำหนดให้ทารกแต่งกายเหมือนเดิมระหว่างการชั่งน้ำหนักทั้ง 2 ครั้ง และชั่งน้ำหนักบนตาชั่งตัวเดิม(ยกตัวอย่างเช่น หากทารกใส่เพียงผ้าอ้อมสำเร็จรูปในการชั่งน้ำหนักก่อนให้นม ระหว่างการให้นมและก่อนการชั่งน้ำหนักหลังให้นมต้องไม่มีการเปลี่ยนเครื่องแต่งกายเช่นเปลี่ยนผ้าอ้อมสำเร็จรูปเป็นตัวใหม่ หรือใส่เสื้อผ้าเพิ่มเติม) เพื่อหาปริมาณน้ำนมที่ทารกได้รับ โดยเทียบน้ำหนักทารกก่อนและหลังรับประทานนมเป็นกรัม (gram) โดยถือว่าผลต่างระหว่างน้ำหนักก่อนและหลังได้รับนมเท่ากับปริมาณน้ำนมที่ได้รับและน้ำหนักที่เพิ่มขั้น 1 กรัม เท่าน้ำน้ำนม 1 มิลลิลิตร (ค่าความหนาแน่นของน้ำนมเท่ากับ 1.03g/ml) และนำมารวบรวมตลอด 24 ชั่วโมง
  - ทำแบบสอบถามเพื่อประเมินคุณภาพชีวิตหลังได้ยาจนครบ 3 วัน โดยใช้แบบทดสอบเครื่องชี้วัดคุณภาพชีวิตขององค์การอนามัยโลกชุดย่อ^(13)^ ฉบับภาษาไทย ฉบับดัดแปลงดังเอกสารที่แนบโดยจะดัดแปลงแบบสอบถามฉบับเต็ม คัดเลือกคำถามเฉพาะข้อที่ประเมินด้านกายและใจเพื่อประเมินคุณภาพชีวิตของอาสาสมัคร
  - สอบถามความพึงพอใจ และติดตามผลข้างเคียงที่เกิดขึ้น การรับประทานนมแม่อย่างเดียว และการรับประทานอาหารอื่นๆร่วมด้วยนอกจากนมแม่ที่ 6 เดือนหลังคลอด
  - บันทึกข้อมูล
  - นำข้อมูลมาวิเคราะห์ทางสถิติ อภิปราย สรุป และนำเสนอผลงานวิจัย
- ขนาดตัวอย่างและการคำนวณ

(Z_1-α/2_+ Z_1-β_)^2^ (σ_1_^2^ + σ_2_^2^/r)

(μ_1 -_ μ_2_)^2^

N =

α = probability of type I error = 0.05

β = 0.2

μ_1_ =mean in controlled group = 135^(6)^

μ_2_ =mean in randomized group = 135 + 40.5 = 175.5

σ_1_= standard deviation in controlled group = 61.5^(6)^

σ_2_ = standard deviation in randomized group = 61.5

- อ้างอิงจากการศึกษาของ Panwara P^(6)^และคณะ คาดหวังว่าจะมีการเพิ่มขึ้นของปริมาณน้ำนมจากการใช้ยาร้อยละ30 เมื่อแทนค่าตามสูตร จะได้จำนวนตัวอย่างในแต่ละกลุ่ม อย่างน้อยเท่ากับ37 ราย (ทั้งหมด 74ราย) และเพิ่มโอกาสการไม่สมัครใจหรือยกเลิกการเข้าร่วมก่อนสิ้นสุดโครงการ (Drop out) ร้อยละ 20คิดเป็นจำนวนกลุ่มละ (N) = 44 คน
- alpha = 0.05
- Power = 80%

1. การวิเคราะห์ข้อมูลและสถิติที่ใช้วิเคราะห์ (Data Analysis and Statistics)

- N = 88
- แสดงผลเป็น Mean, Standard deviation, percent
- สถิติเชิงปริมาณ รายงานผลเป็นค่าเฉลี่ย (mean) และส่วนเบี่ยงเบนมาตรฐาน (standard deviation) ถูกเปรียบเทียบด้วย Student’s T test
- สถิติเชิงคุณภาพ รายงานผลเป็นร้อยละ เปรียบเทียบด้วยChi- square test
- ค่าความเชื่อมั่น ร้อยละ 95 (95% confidence interval)
- ค่า P-value ที่<0.05 ถือว่ามีนัยสำคัญทางสถิติ

1. ข้อพิจารณาด้านจริยธรรม (Ethical Consideration)

- Autonomy อาสาสมัครผู้เข้าร่วมวิจัยมีสิทธิในการตัดสินใจว่าจะเข้าร่วมการวิจัยหรือไม่ด้วยตนเอง โดยการตัดสินใจต้องมีพื้นฐานมาจากการได้รับข้อมูลที่ถูกต้องครบถ้วนและเข้าใจเป็นอย่างดี เกี่ยวกับวิธีดำเนินการวิจัย ความลำบากที่อาจเกิดขึ้น ผลดี และผลเสียที่เกิดขึ้นจากการเข้าร่วมการวิจัยและสามารถตัดสินใจด้วยตัวเองอย่างอิสระ
- Beneficence และNon-Maleficenceผู้เข้าร่วมการวิจัยอาจจะได้รับประโยชน์ในเรื่องของการมีปริมาณน้ำนมสำหรับให้นมบุตรมากขึ้นและงานวิจัยชิ้นนี้จะเป็นการเสริมสร้างองค์ความรู้สำหรับอนาคต ความรู้ที่ได้อาจทำให้เพิ่มตัวเลือกในการใช้ยาเพื่อเพิ่มปริมาณน้ำนมให้หญิงหลังคลอด เป็นการสร้างประโยชน์ในแง่ของการพิสูจน์และค้นพบประสิทธิภาพของยาที่ไม่เคยได้รับการพิสูจน์มาก่อน อาจเกิดความเสี่ยงต่อผู้เข้าร่วมวิจัยคือการสูญเสียเวลาของอาสาสมัครผู้เข้าร่วมการวิจัย ยาชนิดนี้ไม่เคยมีการรายงานผลเสียหรือผลข้างเคียงที่เกิดขึ้น แต่หากดำเนินการวิจัยไปแล้วและพบว่ามีผลเสียเกิดขึ้น ก็จะหยุดการทำวิจัยโดยทันที
- Justice อาสาสมัครที่ถูกคัดเข้ามาร่วมในการวิจัยมีเกณฑ์ในการคัดเลือกเข้า และออกอย่างชัดเจนมีการกระจายความเสี่ยงและผลประโยชน์อย่างเท่าเทียมกัน โดยวิธีสุ่มเข้ากลุ่มศึกษา
- Confidentiality ข้อมูลที่เกี่ยวข้องกับอาสาสมัครผู้เข้าร่วมวิจัยจะถูกเก็บเป็นความลับโดยในแบบบันทึกข้อมูลจะไม่มีข้อมูลที่จะระบุได้ถึงตัวอาสาสมัครได้
- Conflict of Interest ผู้วิจัยไม่มีส่วนเกี่ยวข้อง หรือผลประโยชน์ทับซ้อนใด ๆ จากการทำการวิจัยชิ้นนี้

1. ประโยขน์ที่คาดว่าจะได้รับจากการวิจัย (Expected or Anticipated Benefit gain)

- พิสูจน์ได้ว่ายาแคปซูลใบมะรุมมีสรรพคุณในการเพิ่มปริมาณน้ำนมได้จริง
- เพิ่มตัวเลือกของการรักษาเพื่อเพิ่มปริมาณน้ำนมเพื่อสนับสนุนให้หญิงที่มีความตั้งใจในการให้นมบุตรมีความมั่นใจมากขึ้น

1. อุปสรรค์ที่อาจจะเกิดขึ้น (Challenges)

- ผู้เข้าร่วมวิจัยจำนวนน้อยกว่าที่คำนวณได้
- ไม่ได้รับความร่วมมือในการชั่งน้ำหนักทารกในทุกครั้งที่มีการให้นม

1. ความเสี่ยงที่จะเกิดขึ้นและความรับผิดชอบ (Risk and Investigator’s Responsibility)

- การสูญเสียเวลาของอาสาสมัครผู้เข้าร่วมการวิจัย

1. ระยะเวลาที่ใช้ในการวิจัยตั้งแต่เริ่มต้นจนสิ้นสุดโครงการ (Timeline)

- ตั้งคำถามและหัวข้อการวิจัย 1ธ.ค. 2562 – 31 ธ.ค. 2562
- เขียนโครงร่างการวิจัยและนำเสนอต่อคณาจารย์1ก.ย.2562 –30 พ.ย. 2562
- จัดทำโครงร่างการวิจัยขอคำรับรองจริยธรรมการวิจัย 1 ธ.ค. 2562 – 30 มิ.ย. 2563
- เริ่มดำเนินการขั้นตอนรวบรวมข้อมูลหลังจากโครงการวิจัยได้รับการรับรองจากคณะกรรมการจริยธรรมการวิจัยฯ แล้วจนถึง 30 เม.ย. 2564
- จัดเก็บข้อมูล และวิเคราะห์ทางสถิติ เตรียมพร้อมข้อมูลและเขียนรายงาน 1 เม.ย. 2564 – 30 มิ.ย. 2564
- แก้ไขและเตรียมเสนอรายงานการวิจัย 1 ก.ค. 2564 – 31 ส.ค. 2564

1. การบริหารงานวิจัยและตารางการปฏิบัติงาน (Tabulation of Research Activities and Timeline)


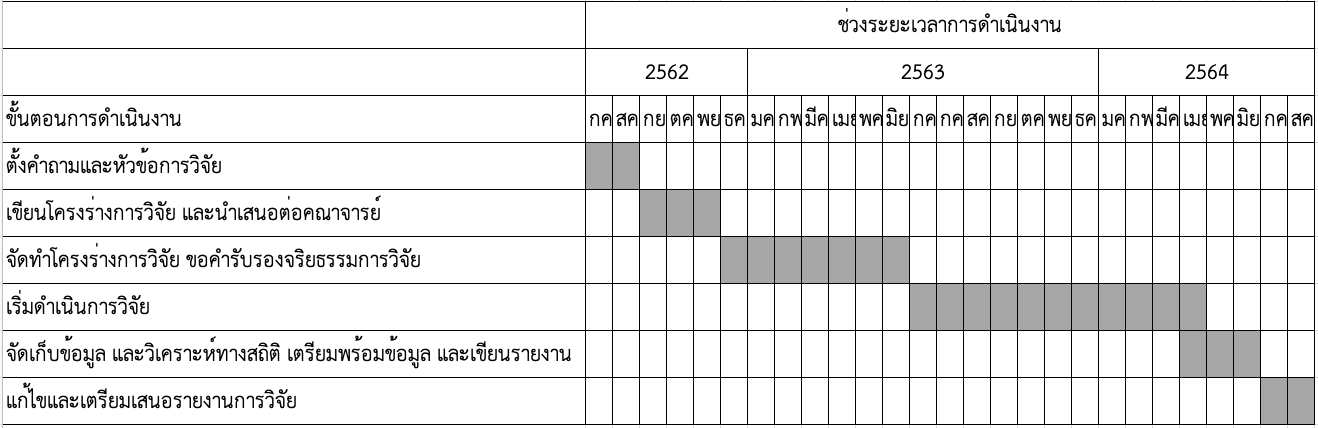


1. สถานที่ทำวิจัย (Venue of the Study)

- โรงพยาบาลจุฬาลงกรณ์ สภากาชาดไทยอาคารภูมิสิริ หอผู้ป่วย 17A, 22A, 22B, 22C

1. งบประมาณรายจ่ายของโครงการวิจัย

| รายการ | จำนวนเงิน |
| --- | --- |
| ค่ายาแคปซูลใบมะรุม เม็ดละ 2 บาท ใช้วันละ 2 เม็ด ทั้งหมด 3 วัน (จำนวน 88 คน) | 1056 |
| ค่าตอบแทนอาสาสมัคร คนละ 300 บาท (จำนวน 88 คน) | 26400 |
| ค่าเครื่องชั่งน้ำหนักระบบดิจิทัล เครื่องละ 2000 บาท ทั้งหมด 4 เครื่อง | 8000 |
| ค่าใช่สอยอื่นๆ |  |
| - ค่าใช้สอยสำนักงาน เช่น การจัดทำรายงาน ค่าถ่ายเอกสาร จัดส่งเอกสารส่งแฟกซ์ | 5000 |
| - ค่าใช้จ่ายในการหาข้อมูลและตีพิมพ์ | 3000 |
| รวม | 43456 |

1. งบประมาณในการทำวิจัย

- วางแผนการขอทุนจากกองทุนรัชดาภิเษกสมโภช

References

1. Maharlouei N, Pourhaghighi A, Raeisi S H, Zohoori D, Lankarani KB. Factors affecting exclusive breastfeeding, using adaptive LASSO regression. Int J Community Based Nurs Midwifery 2018;6(3):260–271.
2. SitiN, ImamiN R, Hayuni R.Breastfeeding self-efficacy as a dominant factor affecting maternal breastfeeding satisfaction. BMC Nurs 2019;18(1):30-37.
3. Janet F, Jon A, Amie S, Alex B, Cindy G, David S.Women’s Use and Self-Prescription of Herbal Medicine duringPregnancy: An Examination of 1,835 Pregnant Women. Women’s Health Issue 2015;25(4):396-402.
4. OthmanN, R A CLamin, C N Othman.Exploring Behavior on the Herbal Galactagogue Usageamong Malay Lactating Mothers in Malaysia. ProcediaSocial Behav Sci 2014;153:199-208.
5. Antonia Z, Jennifer G, Lea S. Use of Herbals as Galactagogues. JPharmPract 2012;25(2):222-231.
6. Panwara P, Kasem R,Wipada L, Maysita S, and Pawin P.The Effect of Ginger on Breast Milk Volume in the Early Postpartum Period: A Randomized, Double-Blind Controlled Trial.Breastfeed Med 2016;11(7):361-365.
7. Raguindin PF, Dans LF, King JF.Moringa oleifera as a Galactagogue. Breastfeed Med2014; 9(6):323-4.
8. Estrella CP, Blas J, David GZ, Taup MA.A double-blind, randomized controlled trial on the use of malunggay (Moringaoleifera) for augmentation of the volume ofbreastmilk among non-nursing mothers of preterm infants.Philipp J Pediatr 2000;49(1):3-6.
9. EspinosaK,Criselda L. A randomized controlled trial on the use of Malunggay (Moringaoleifera) for augmentation of the volume of breastmilk among mothers of term infants. Fil Fam Phys 2005;43(1):26–33.
10. Sidney J. Stohs, Michael J. Hartman. Review of the Safety and Efficacy of Moringa oleifera. Phytother Res 2015;29(6):796-804.
11. Butte NF, Wong W, Patterson BW, et al. Human milk intake measured by administration of deuterium oxide to the mother: A comparison with the test-weighing technique. Am J ClinNutr1988; 47(5):815–821.
12. Krishanu S, Atmatrana T M. Efficacy and tolerability of a novel herbalformulation for weightmanagement in obesesubjects: a randomized double-blind placebocontrolled clinical study. LipidsHealth Dis 2012;11:122.
13. Mahin K, Mojgan M, Fatemeh R, Nasrin G.Quality of Life Predictors in Breastfeeding Mothers Referred to Health Centers in Iran. Int. J. Women's Health ReprodSci2018;6(1):84-89.
