# Supplementary material for: The effect of Moringa oleifera capsule in increasing breastmilk volume in early postpartum patients: A double-blind, randomized controlled trial
Source: PLoS One. 2021 Apr 6;16(4):e0248950. doi: 10.1371/journal.pone.0248950 (PMC8023461; doi:10.1371/journal.pone.0248950)
Supplement: S2 File — (DOC) [file pone.0248950.s002.doc]

**Research Proposal**

1. Proposal Title

Effect of Moringa oleifera leaf capsule in Increasing Breast Milk Volume in Early Postpartum Patients, A Double blind, Randomized Controlled Trial

1. Investigators

Siraphat Fungtammasan M.D, Vorapong Phupong, M.D.

Department of Obstetrics and Gynecology, Faculty of Medicine, Chulalongkorn University, Rama IV Road, Pathumwan, Bangkok 10330, Thailand

1. Rationale

- Breastmilk is the best food for the baby since after birth to 6 months. It is safe, clean and contains antibodies which protect them against common illnesses. It also contains good nutrients and energy for the baby especially in the first month of life. Breastfeeding provides the physiological and health benefits for both the mother and the baby. One of the obstacles for breast feeding is inadequate breastmilk volume. ^(1),(2)^ Various methods have been used to increase the volume of breastmilk. Galactagogue herbs have been used by breastfeeding mothers who have breastmilk problem to increase the volume of breastmilk. Galactagogue herbs have been used instead of synthetic medication because of safety and fewer side effects. ^(3),(4)^ Galactagogue herbs that have been used to increase breastmilk volume are ginger, long pepper, [finger root](https://dict.longdo.com/search/fingerroot) and Moringa oleifera ^(5-9)^ However, there have been a few studies of galactagogue herbs in increasing breastmilk volume. The authors would like to conduct this study to find the effect of Moringa oleiferaleaf capsule in increasing breastmilk volume

1. Literature Review

- Raguindin ^(7)^ from Philippines reported in year 2014. They reported that Moringa can result in a significant increase in breastmilk volume by increasing prolactin levels. Prolactin levels were higher in women who received Moringa that those who received placebo.
- Janet et al.^(3)^ from Australia and Othman et al^(4)^ from Malaysia found that many women consumed herbal medicine during pregnancy. The self-prescription of herbal medicine by pregnant women was of concern owing to potential safety issues. Most mothers consumed herbal galactagogue due to insufficient milk ejection.
- Estrella et al^(8)^ in year 2000 performed a Randomized Controlled Trial on the use of Moringa oleifera for augmentation of the volume of breastmilk among non-nursing mothers of preterm infants. The dosage was 250 mg orally every 12 hours starting from postpartum day 3 to 5. The mothers were asked to pump their breasts using a standardized breast pump from day 1 to day 5 postpartum. They found that there was a trend towards increased milk production among those on Moringa oleifera leaves on day 3, 4 and 5 without side effects.
- Espinosa-Kuo ^(9)^ in year 2005 performed a single blind randomized controlled trial on the use of Moringa oleifera for augmentation of the volume of breastmilk among mothers of term infants. The dosage was 350 mg, 2 capsules daily starting from postpartum day 3 to 10. They found that the amount of breastmilk per day was produced by subjects who took Moringa oleifera than those who took the placebo. However. This was not statistically significant.
- Paritakul et al^(6)^ performed a Randomized Controlled trial of ginger on breast milk volume in the early postpartum period. Women who deliver a term baby were randomly assigned to receive dried ginger or placebo for 7 days postpartum. Breast milk volume was measured on third day postpartum using test weight method for a period of 24 hours and on seventh day postpartum using 1 hour milk production. They also compared the third day serum prolactin level between the two groups. They found that women in the ginger group have higher milk volume than the placebo group However, the seventh day milk volume in the ginger group does not differ from the placebo group. The mean serum prolactin levels were similar in both groups and no side effect was reported in the study.
- Stohs et al^(10)^in year 2015 reviewed the safety and efficacy of Moringa oleifera. Leaf extracts exhibit the greatest antioxidant activity, and various safety studies in animals involving aqueous leaf extracts indicate a high degree of safety. No adverse effects were reported in association with human studies. Powdered whole leaf preparations of M. oleifera have demonstrated anti-hyperglycemic (antidiabetic) and anti-dyslipidemic activities. Various animal studies have assessed the general safety of extracts, and have demonstrated a very high degree of safety. In rats, the Moringa oleifera leaf extract was shown to be genotoxic based on blood cell analysis at the 3000 mg/kg dose.
- Butte et al in year 1988^(11)^  performed a study “Human-milk intake measured by administration of deuterium oxide to the mother: a comparison with the test-weighing technique”. A comparison was made between the dose-to-the-mother deuterium-dilution method and the conventional test-weighing technique for determining human-milk intake. After administration of 2H to the mothers human milk and infant urine were sampled over 14 d and analyzed for 2H:1H ratios by gas-isotope-ratio mass spectrometry. Infant total body water was determined by 18O dilution. The test-weighing procedure was conducted for 5 d consecutively. The mean difference between the two methods was not significantly different from 0. The 2H-dilution and test-weighing techniques provide similar estimates of human-milk intake.

1. Objectives

Primary objective:

- To compare the breastmilk volume between postpartum mothers who receive Moringa oleifera leaf capsule with those receiving placebo.

Secondary objectives:

- To compare satisfaction, quality of life, and side effects of Moringa oleifera leaf between postpartum mothers who receive Moringa oleifera leaf capsule with those receiving placebo.

1. Hypothesis

Primary research question:

- Can Moringa oleifera leaf capsule increase breastmilk volume in postpartum mothers?

Secondary research questions:

- Can Moringa oleifera leaf capsule increase satisfaction, and quality of life?
- Does Moringa oleifera leaf capsule cause any side effects?

1. Keywords

- Lactation
- Galactogogue
- Moringa oleifera
- Drumstick tree
- Breast milk volume
- Herbal medicine

1. Research design

- Randomized Double blind Placebo Controlled Trial

1. Research Methodology

- Population: postpartum women who intend to breastfeed
- Target Population: postpartum women age at least 18 years, delivered at King Chulalongkorn Memorial Hospital, Faculty of Medicine, Chulalongkorn University who intend to breastfeed
- Control Population: postpartum women age at least 18 years, delivered at King Chulalongkorn Memorial Hospital, Faculty of Medicine, Chulalongkorn University who intend to breastfeed, and receive placebo
- Inclusion Criteria:
  - postpartum women age at least 18 years who intend to breastfeed
- Exclusion Criteria:
  - Postpartum women with contraindication to breastfeeding such as
    - Substance abuse: amphetamine, cocaine, heroin, marijuana, phencyclidine, alcohol, smoking
    - Baby with galactosemia
    - HIV infection, Human T cell leukemia virus, Infectious mononucleosis , Ebola virus,Marburg virus, Lassa virus, dengue virus, adenoviruses
    - Untreated tuberculosis
    - Current of these medications:
      - Cyclophosphamide Cyclosporine Doxorubicin Methotrexate
      - Copper 64 (64Cu), Gallium 67 (67Ga), Indium 111 (111In), Iodine 123 (123I),Iodine 125 (125I), Iodine 131 (131I), Radioactive sodium, Technetium 99m (99mTc), Macroaggregates, sodium pertechnetate (99mTcO4)
  - Postpartum women with condition that cannot breastfeed: critical condition, unstable vital signs, postpartum hemorrhage
  - women with known allergy to Moringa oleifera
  - women whose baby need phototherapy
  - women with insufficient glandular tissue or breast surgery
  - women with a history of infertility
  - women with hypothyroidism
  - women with twins or higher order births, premature infants and infants with sucking problems or structural oral anomalies that can affect sucking (eg. tongue tie, birth asphyxia, clefts, etc.)
- Informed consent process
  - Participants received the management from doctors, nurses and staffs who do not involve in the study. Thus, they can decide to participate in the study by themselves without pressure. Participants, who are interested in the study, will receive information sheet of the study. They can ask the questions and spend time before making the decision. They sign the consent form before enrolling into the study.
- Methodology
  - Write research proposal and submit to Ethical committee of Faculty of Medicine, Chulalongkorn University
  - Pregnant women aged 18 years or more and gestational age 37 weeks or more who intend to breastfeed will be invited to join this study. Recruitment is done and consent is obtained before delivery. Randomization will be done after delivery.
  - Enroll the participants who meet the inclusion criteria from postpartum women age at least 18 years, delivered at King Chulalongkorn Memorial Hospital, Faculty of Medicine, Chulalongkorn University who intend to breastfeed. Participants who have exclusion criteria were excluded.
  - Participants will receive the study information and can ask the questions regarding the study.
  - Participants can make the decision by themselves and sign informed consent.
  - Participants will be random allocation by block of 4 techniques into treatment and control group.
  - Study data will be recorded in case record form and data entry in computer by research assistance.
  - Participants will be random into treatment and control group. Treatment group receive Moringa oleifera leaf capsule (450 mg) 1 capsule before breakfast and dinner for 3 days.^(12)^ Control group receive placebo capsule for 3 days. Participants will take their first capsule at first 6 hours of birth. The drugs and placebo will be prepared prior to the study by a pharmacist who is not involved in the study. As for the placebo capsule, there will be no drug in the capsule. As soon as the participant meets the inclusion criteria, the nurses will proceed to select a sequentially numbered opaque envelope. The opaque envelopes are sequentially labeled and will contain 6 capsules of Moringa oleifera leaves powder or placebo (identical in size, shape and color). To ensure randomization, each envelope will be distributed in a sequential numerical order. Both the health care providers and the participants will be masked to the treatment assignment. Treatment will be started at postpartum. Treatment assignment will not be revealed until data collection at 6 months is completed.
  - Moringa oleifera leaves powder (450mg per capsule) is distributed by Ouay Un Osoth, Thailand. This standard is certified by GMPPIC/S(Good Manufacturing Practice Pharmaceutical Inspection Cooperation Scheme from Thai FDA (code no. 73-1-49541-1-0015)
  - Breastmilk volume will be recorded at day 3 postpartum (48-72 hours postpartum). The weighing method will be used. The weighing procedure will start at 48 hours after delivery in all women. The nurse will weigh the infant fully clothed before and after each feeding using an electronic weight scale (Camry ER 7210, accurate to 5 g) for the period of 24 hours. The volume of the breastmilk will be evaluated. The sum of the weight difference in gram will be converted into the volume of the breastmilk in milliliter (1 g = 1mL). This method is comparable with the measurement of the volume of the breastmilk based on deuterium oxide dilution technique from a previous study^(11)^  .
  - Participants answer the quality of life questionnaire at 3 days postpartum by using WHOQoL-BREF ^(13)^ Thai version.
  - Record participants’ satisfaction, side effects, exclusive breast feeding and any breast feeding at 6 months postpartum.
  - Data entry in computer by research assistance.
  - Statistical analysis and present the result of the study.
- Sample size calculation

N = (Z_1-α/2_+ Z_1-β_)^2^ (σ_1_^2^ + σ_2_^2^/r)

(μ_1 -_ μ_2_)^2^

α = probability of type I error = 0.05

β = 0.2

μ_1_ =mean in controlled group = 135^(6)^

μ_2_ =mean in randomized group = 135 + 40.5 = 175.5

σ_1_= standard deviation in controlled group = 61.5^(6)^

σ_2_ = standard deviation in randomized group = 61.5

- From Paritakul et al’s study^(6)^ , we expect the 30% increased breastmilk volume. A minimum of 37 women in each group were required to detect statistical difference (α=0.05, β=0.2) with adjustments for a drop out rate of 20%, Thus, total 44 women per group were required in this study.
- alpha = 0.05
- Power = 80%
- Total N = 88

1. Data Analysis and Statistics

- Data were presented as mean, standard deviation, percent
- Independent t-test was used to compare continuous variables, and Mann-Whitney U test was used for nonparametric variables.
- Chi-square test and Fisher-exact test were used to compare categorical variables.
- A p value <0.05 was considered statistically significant.
- Analysis of the trial was conducted in intent-to-treat (ITT) analysis.

1. Ethical Consideration

- Autonomy: Participants themselves can decide whether to participate in the study. The decision depends on the information of the study, risk and benefit.
- Beneficence and non-maleficence: participants will have the benefit of increasing breastmilk volume. The risk for the participants is the wasting time for the study. No adverse effects of Moringa oleifera have been reported in association with human studies. If the side effects occur, participants will receive the treatment without cost.
- Justice: participants will be included into the study and excluded according to criteria. They will be random into each group by block of 4 techniques. The distribution of costs and benefits to *potential* research participants is fair and equally.
- Confidentiality: anonymous data will be recorded in case record form. Personal or identifiable information of the participants will not be disclosed to others.
- Conflict of Interest: The researchers declare that they have no competing interests.

1. Expected or Anticipated Benefit gain

- To proof that Moringa oleifera leaf capsule can increase breastmilk volume in postpartum mothers.
- Give the alternative management to increase the breastmilk volume. This will increase the percentage of exclusive breastfeeding.

1. Obstacles and challenges

- Small number of participants
- Without cooperation for weighing the baby

1. Risk and Investigator’s Responsibility

- Participants have to spend the time for participate the study.

1. Timeline

- Research question development 1 Jul 2019 – 31 Aug 2019
- Proposal development 1 Sep 2019 – 30 Nov 2019
- Ethical committee submission 1 Dec 2019 – 30 Jun 2020
- Study will start after Ethical Committee approve
- Data entry and statistical analysis 1 Apr 2021– 30 Jun 2021
- Present the outcomes and manuscript development 1 Jul 2021 – 31 Aug 2021

1. Tabulation of Research Activities and Timeline

|  | 2019 | | | | | | 2020 | | | | | | | | | | | | 2021 | | | | | | | |
| --- | --- | --- | --- | --- | --- | --- | --- | --- | --- | --- | --- | --- | --- | --- | --- | --- | --- | --- | --- | --- | --- | --- | --- | --- | --- | --- |
|  | Jul | Aug | Sep | Oct | Noc | Dec | Jan | Feb | Mar | Apr | May | June | Jul | Aug | Sep | Oct | Noc | Dec | Jan | Feb | Mar | Apr | May | June | Jul | Aug |
| Research question development |  |  |  |  |  |  |  |  |  |  |  |  |  |  |  |  |  |  |  |  |  |  |  |  |  |  |
| Proposal development |  |  |  |  |  |  |  |  |  |  |  |  |  |  |  |  |  |  |  |  |  |  |  |  |  |  |
| Apply for IRB authorization |  |  |  |  |  |  |  |  |  |  |  |  |  |  |  |  |  |  |  |  |  |  |  |  |  |  |
| Proceed the study |  |  |  |  |  |  |  |  |  |  |  |  |  |  |  |  |  |  |  |  |  |  |  |  |  |  |
| Data entry and analysis |  |  |  |  |  |  |  |  |  |  |  |  |  |  |  |  |  |  |  |  |  |  |  |  |  |  |
| Present the final outcome and  manuscript development |  |  |  |  |  |  |  |  |  |  |  |  |  |  |  |  |  |  |  |  |  |  |  |  |  |  |

1. Venue of the Study

- King Chulalongkorn Memorial Hospital, Faculty of Medicine, Chulalongkorn University: Pumisirimangkaranusorn Building ward no. 17A, 22A, 22B, 22C

1. Budget

|  | Amount |
| --- | --- |
| Moringa oleifera leaf capsule (2 Baht/ capsule * 6 * 88 cases) | 1056 Baht |
| Compensation (300 Baht/participant * 88 cases) | 26400 Baht |
| electronic weight scale (2000 Baht/scale * 4) | 8000 Baht |
| Office expense | 5000 Baht |
| Expense for data collection | 3000 Baht |
| Total expense | 43456 Baht |

1. Funding

- Internal research grant: Ratchadaphiseksompotch, Faculty of Medicine, Chulalongkorn University

20. References

1. Maharlouei N, Pourhaghighi A, Raeisi S H, Zohoori D, Lankarani KB. Factors affecting exclusive breastfeeding, using adaptive LASSO regression. Int J Community Based Nurs Midwifery 2018;6(3):260–271.
2. SitiN, ImamiN R, Hayuni R.Breastfeeding self-efficacy as a dominant factor affecting maternal breastfeeding satisfaction. BMC Nurs 2019;18(1):30-37.
3. Janet F, Jon A, Amie S, Alex B, Cindy G, David S.Women’s Use and Self-Prescription of Herbal Medicine duringPregnancy: An Examination of 1,835 Pregnant Women. Women’s Health Issue 2015;25(4):396-402.
4. OthmanN, R A CLamin, C N Othman.Exploring Behavior on the Herbal Galactagogue Usageamong Malay Lactating Mothers in Malaysia. ProcediaSocial Behav Sci 2014;153:199-208.
5. Antonia Z, Jennifer G, Lea S. Use of Herbals as Galactagogues. JPharmPract 2012;25(2):222-231.
6. Panwara P, Kasem R,Wipada L, Maysita S, and Pawin P.The Effect of Ginger on Breast Milk Volume in the Early Postpartum Period: A Randomized, Double-Blind Controlled Trial.Breastfeed Med 2016;11(7):361-365.
7. Raguindin PF, Dans LF, King JF.Moringa oleifera as a Galactagogue. Breastfeed Med2014; 9(6):323-4.
8. Estrella CP, Blas J, David GZ, Taup MA.A double-blind, randomized controlled trial on the use of malunggay (Moringaoleifera) for augmentation of the volume ofbreastmilk among non-nursing mothers of preterm infants.Philipp J Pediatr 2000;49(1):3-6.
9. EspinosaK,Criselda L. A randomized controlled trial on the use of Malunggay (Moringaoleifera) for augmentation of the volume of breastmilk among mothers of term infants. Fil Fam Phys 2005;43(1):26–33.
10. Sidney J. Stohs, Michael J. Hartman. Review of the Safety and Efficacy of Moringa oleifera. Phytother Res 2015;29(6):796-804.
11. Butte NF, Wong W, Patterson BW, et al. Human milk intake measured by administration of deuterium oxide to the mother: A comparison with the test-weighing technique. Am J ClinNutr1988; 47(5):815–821.
12. Krishanu S, Atmatrana T M. Efficacy and tolerability of a novel herbalformulation for weightmanagement in obesesubjects: a randomized double-blind placebocontrolled clinical study. LipidsHealth Dis 2012;11:122.
13. Mahin K, Mojgan M, Fatemeh R, Nasrin G.Quality of Life Predictors in Breastfeeding Mothers Referred to Health Centers in Iran. Int. J. Women's Health ReprodSci2018;6(1):84-89.
