# Supplementary material for: The effect of Moringa oleifera capsule in increasing breastmilk volume in early postpartum patients: A double-blind, randomized controlled trial
Source: PLoS One. 2021 Apr 6;16(4):e0248950. doi: 10.1371/journal.pone.0248950 (PMC8023461; doi:10.1371/journal.pone.0248950)
Supplement: S1 Table — (DOC) [file pone.0248950.s005.doc]

**S1 Table. Baseline characteristics.**

| Baseline characteristics | Moringa oleifera group (n=44) | Placebo group (n=44) | P value |
| --- | --- | --- | --- |
| Maternal age (years) |  |  |  |
| Gravida |  |  |  |
| Primigravida |  |  |  |
| Multigravida |  |  |  |
| Parity |  |  |  |
| Primiparity |  |  |  |
| Multiparities |  |  |  |
| BMI (kgs/m^2^) |  |  |  |
| Total weight gain (kgs) |  |  |  |
| Vital signs  Blood pressure (mmHg)  Pulse rate (beats/minute)  Body temperature (°C) |  |  |  |
| GA at delivery (weeks) |  |  |  |
| Route of delivery   - Vaginal route - Cesarean section |  |  |  |
| Labor medication |  |  |  |
| - spinal bupivacaine  - pethidine  - xylocaine  - |  |  |  |
| Amount of intravenous fluid (ml) |  |  |  |
| Baby gender  Male  Female |  |  |  |
| Birth weight (grams) |  |  |  |
| Time of first sucking (hours after delivery) |  |  |  |
| Number of breastfeeding per day |  |  |  |
| Supplemental feeds |  |  |  |
| Do you think you got the placebo or the intervention?  - placebo  - intervention |  |  |  |

Data presented as mean + SD, n (%) or median (interquartile range).
