# Supplementary material for: The effect of Moringa oleifera capsule in increasing breastmilk volume in early postpartum patients: A double-blind, randomized controlled trial
Source: PLoS One. 2021 Apr 6;16(4):e0248950. doi: 10.1371/journal.pone.0248950 (PMC8023461; doi:10.1371/journal.pone.0248950)
Supplement: S2 Table — (DOCX) [file pone.0248950.s006.docx]

**S2 Table. Breast milk volume, satisfaction, quality of life and side effects.**

| Result | Moringa oleifera group (n=44) | Placebo group (n=44) | P value |
| --- | --- | --- | --- |
| Breast milk volume (ml) |  |  |  |
| Day 1 |  |  |  |
| Day 2 |  |  |  |
| Day 3 |  |  |  |
| Participants noticed their breasts were full (Yes/No) |  |  |  |
| When did participants feel breast fullness? |  |  |  |
| Satisfaction |  |  |  |
| Quality of life |  |  |  |
| Side effect   - constipation - nausea/vomiting - diarrhea - heartburn - hypotension - hypoglycemia - others   newborn   - hypotension - hypoglycemia |  |  |  |

Data presented as mean + SD or n (%).
